# When austerity becomes a public health threat: the case of Ecuador

**DOI:** 10.3389/fpubh.2026.1749759

**Published:** 2026-01-22

**Authors:** Esteban Ortiz-Prado, Jorge Vasconez-Gonzalez, Juan S. Izquierdo-Condoy

**Affiliations:** One Health Research Group, Faculty of Health Science, Universidad de Las Americas, Quito, Ecuador

**Keywords:** austerity, public health, health budget, health disparities, Ecuador

To the Editor,

As a public health researcher working in Ecuador, I have witnessed firsthand the devastating consequences of sustained disinvestment in the health sector. Between 2021 and 2025, Ecuador's public health budget regressed to pre-pandemic levels, with an alarming USD 1.3 billion cut in 2024 alone—falling from an initial allocation of USD 4.2 billion to just USD 2.7 billion executed (1). For 2025, the budget stands at merely USD 2.738 billion (1). This regression not only violates the constitutional mandate to progressively allocate at least 4% of the GDP to health but also undermines fragile progress toward Universal Health Coverage.

These cuts are not merely accounting decisions—they can be fatal. Health facilities in remote regions face chronic shortages of essential medicines and diagnostic, therapeutic, and treatment equipment (2). Budget allocations for pharmaceuticals and medical devices declined from USD 447 million in 2023 to USD 348 million in 2025 (1). Preventive programs—including maternal and child health, intercultural health services, mental health, reproductive health, and non-communicable disease control—have been suspended, with no new public health initiatives formulated since the current administration took office. In structurally marginalized Amazonian communities like Taisha, emergency transport, and outreach services have collapsed due to fuel shortages, exacerbating outbreaks such as leptospirosis, which surged to 937 reported cases nationwide in 2025, up from 663 in 2023 (3–6).

This pattern is not new. Austerity measures, often introduced under the guise of fiscal responsibility, have historically worsened health outcomes in other contexts, including Europe and Latin America, disproportionately affecting the most vulnerable populations (7–9). In Ecuador, under-execution has further compounded the crisis: by mid-2025, only 4% of the USD 276 million allocated for medical equipment had been executed (1). Meanwhile, the centralized public procurement system—intended to ensure medication availability—has deteriorated, and essential hospital services such as cleaning, food provision, and laundry have faced an important budget reductions, severely impairing hospital operations and even causing food shortages for patients and staff (10).

This reality must be understood within the broader context of the Global South, where debt, structural adjustment, and declining public investment in social protection systems converge to produce what some scholars have termed “biological sub-citizenship” (11). In Ecuador, more than 2,500 health sector workers were laid off in 2019. In 2024, an additional 500 workers from Social Security hospitals were dismissed, and the Ministry of Health has seen four different ministers in under 2 years, one of whom was not a health professional (12–15). Governance instability, under-execution of approved budgets, and technocratic reforms that prioritize efficiency over equity mirror patterns observed in other low- and middle-income countries facing post-pandemic fiscal contractions (16).

Ecuador's democratically elected administration, which ran on a platform of modernization and efficiency, inherited functional health tools and infrastructure from previous governments. However, its decision to prioritize fiscal tightening over health investment has already begun to reverse critical public health gains. When centrist or market-oriented governments fail to guarantee essential protections such as access to healthcare, their claims to superior governance collapse—not in rhetoric, but in measurable outcomes.

The Global South cannot afford a return to austerity. Robust evidence shows that health equity deteriorates under such regimes, especially when social protection systems are weakened or dismantled (17, 18). Ecuador has an opportunity to break this cycle. To do so, it must treat health not as a cost but as a strategic investment. This entails restoring the health budget, ensuring full execution of funds, and prioritizing primary and preventive care over reactive, high-cost interventions. Otherwise, what we are witnessing is not a fiscal reform it is a slow-motion public health catastrophe that is both preventable and unjust.
